# Is damage to the ventromedial prefrontal cortex associated with impaired empathy? A neuropsychological study using an empathy motivation task

**DOI:** 10.1093/scan/nsag072

**Published:** 2026-09-02

**Authors:** Julian A Scheffer, Justin Reber, C Daryl Cameron, Daniel Tranel

**Affiliations:** Department of Psychology & Centre for Brain and Mind, University of Western Ontario, London, Ontario, N6A 3K7, Canada; Department of Neurology, University of Iowa, Iowa City, IA 52242, United States; Department of Neurology, University of Iowa, Iowa City, IA 52242, United States; Department of Psychology and Rock Ethics Institute, The Pennsylvania State University, University Park, PA 16802, United States; Department of Neurology, University of Iowa, Iowa City, IA 52242, United States

**Keywords:** affective empathy, cognitive empathy, free-choice, lab behavior

## Abstract

Patients with damage to the ventromedial prefrontal cortex (vmPFC) classically develop personality disturbances, emotion dysregulation, and social decision-making deficits that have been likened to psychopathic tendencies (“pseudopsychopathy”). These acquired deficits are thought to entail empathy impairments, viz., identifying other people’s emotional experiences and caring about their well-being. However, these impairments may be due to either low motivation to empathize or impaired ability to empathize accurately. We examined whether patients with vmPFC lesions (who have been shown previously to have major deficits in empathy accuracy) would exhibit low empathy motivation. We conducted a lesion group comparison study to assess motivation to empathize. The results showed that damage to the vmPFC was not associated with low empathy motivation (including avoidance of affective or cognitive forms of empathy). Specifically, patients with vmPFC lesions did not avoid empathy any more so than comparison groups (participants with damage outside of the vmPFC or participants with no brain damage). Our findings suggest that patients with vmPFC damage may have preserved empathy motivation, at least in an off-line, laboratory task setting, despite their real-world impairments with deploying empathy. More research is needed to examine whether patients with vmPFC lesions would exhibit normal empathy motivation in everyday contexts.

## Introduction

The ventromedial prefrontal cortex (vmPFC) has been strongly linked to empathy, defined as identifying, sharing, and understanding other people’s emotional experiences. Altered morphology and functioning in the vmPFC have been consistently associated with empathy impairments. Numerous fMRI studies have consistently found that participants with callous-unemotional or psychopathic traits have reduced BOLD activation in the vmPFC in response to fearful faces or other emotional stimuli (Blair 2007, Yang *et al.* 2008). Additionally, smaller vmPFC volume has been consistently associated with clinical psychopathy (both in overall diagnosis and individual traits) and with conduct disorder, conditions marked by a lack of empathy (Blair 2005, 2007, Sterzer *et al.* 2007, Huebner *et al.* 2008, Yang *et al.* 2008, Ermer *et al.* 2012, Passamonti *et al.* 2012, Cope *et al.* 2014, Vieira *et al.* 2015). Case studies of patients with vmPFC lesions revealed profound changes in personality and socioemotional function (including ability to empathize) in the setting of well-preserved intelligence, memory, and other cognitive abilities. We turn to some of the most prototypical of such case studies.

### Case studies of empathy ability in patients with vmPFC lesions

Phineas Gage, who was the victim of an explosion resulting in a tamping iron being hurled through his face, skull, and brain, was previously described as a “responsible, intelligent, and socially well-adapted individual, a favorite with peers and elders” (Harlow 1868). Following the accident, he showed no impairments of movement, speech, memory, or intelligence. However, his ability to maintain social conventions was radically altered, as he appeared no longer concerned with the feelings and perspectives of others or with honoring his responsibilities, relationships, and commitments to others (Harlow 1869). Contemporary case studies like Gage’s have also been reported. Patient EVR (Eslinger and Damasio 1985) displayed changes in socioemotional function following resection of a large orbitofrontal meningioma resulting in damage to the mesial and ventral regions of both his left and right frontal lobes. Prior to tumor resection, he was happily married, the father of two well-adjusted children, and a leader in his community. However, after surgery EVR developed profound personality changes, socioemotional impairments, and a loss of respect for social conventions (Eslinger and Damasio 1985). These and other case studies implicate vmPFC lesions with a shift in personality and a lack of regard for the feelings of other people, viz., impaired empathy (Anderson *et al.* 1999, 2006).

### Lesion comparison studies examining the vmPFC’s contribution to empathy ability

Lesion group comparison studies have shown that selective damage to the vmPFC is associated with empathy impairments. For example, patients with vmPFC lesions have deficits in theory of mind (Shamay-Tsoory *et al.* 2003) and evaluating their own mental states to be able to interpret or predict the mental states of others (Shamay-Tsoory *et al.* 2003, 2005, 2009). They also often exhibit impairments in emotion recognition (Grattan *et al.* 1994, Leigh *et al.* 2013, Hillis 2014), having difficulty identifying these from other people’s facial expressions, particularly for negative emotions such as fear and disgust (Adolphs *et al.* 1999, Heberlein *et al.* 2008, Vandekerckhove *et al.* 2014). Patients with vmPFC lesions also have deficits identifying emotion from vocal prosody (Hornak *et al.* 1996), as well as reductions in spontaneous perspective-taking and affective theory of mind (Shamay-Tsoory and Aharon-Peretz 2007, Shamay-Tsoory *et al.* 2009). Damage to the vmPFC has also been associated with reduced physiological arousal when viewing images of others in pain or discomfort (Damasio *et al.* 1990) and when preparing to make hypothetical moral choices that would harm others (Moretto *et al.* 2010). Thus, patients with vmPFC lesions exhibit notable impairments with identifying and recognizing the emotional experiences of others, as well as with generating the physiological arousal that associates with emotion sharing and concern.

There is additional evidence that empathy impairments of patients with vmPFC lesions extend beyond perceptual tasks such as emotion identification and theory of mind into behavioral expressions of empathy. Individuals with vmPFC lesions were less generous to others on neuroeconomic tasks such as the Dictator Game, although generosity may not always originate from empathy (Krajbich *et al.* 2009, Koenigs *et al.* 2010). In one evocative study that manipulated empathy to replicate how it manifests in everyday life, participants overheard a study confederate discussing a personal tragedy before playing the Ultimatum Game. Comparison participants allocated more money to partners who they believed were suffering; however, patients with vmPFC damage did not (Beadle *et al.* 2018). As such, previous case studies and lesion research have highlighted that acquired vmPFC damage causes severe impairments with everyday social functioning including empathy and social behavior, while potentially showing preserved functional ability in other domains (e.g. on intellectual ability, memory, and language; Grafman *et al.* 1990, Anderson *et al.* 1991). Laboratory paradigms, which aim to replicate real-world situations, have elicited these types of empathy impairments in patients with vmPFC damage.

### Examining the contribution of the vmPFC to empathy motivation

Though plenty of evidence supports the position that the vmPFC is important in empathy ability and its functioning, recent theories and perspectives have proposed a critical and separate focus on motivation to empathize (Keysers and Gazzola 2014, Cameron *et al.* 2022). Following the logic of Keysers and Gazzola (2014), one can think about capacity as a latent upper boundary on empathy, and previous understanding of vmPFC-empathy studies implies a capacity-based framing rather than a motivational one. Here, we conceptualize empathy motivation as an increased propensity or willingness to put oneself into empathy-eliciting mindsets or situations.

The vmPFC has been documented to be involved in integrating value and reward in decision-making, with earlier work showing that patients with damage to these regions show issues with integrating satiation into their decisions to consume food (Reber *et al.* 2017). Lesion mapping research has found that impairments in value-based decision making (e.g. on the Iowa Gambling Task) are associated with lesions to a network involving the vmPFC (Gläscher *et al.* 2012). Moreover, the vmPFC is involved in the exertion of effort for prosocial motivation and behavior (Lockwood *et al.* 2024, Gueguen *et al.* 2025) and in the anticipation of mental effort exertion (Hogan *et al.* 2019).

In the context of empathy and decision making, the vmPFC may support the integration of value from previous experiences of empathy and updating the anticipated value of empathy engagement. For example, if rewards are encountered (e.g. increasing trust and positivity from others) or costs (e.g. emotional exploitation and burnout) that become more prominent with choosing to approach or avoid empathy (Zaki 2014, Shamay-Tsoory and Hertz 2022), it may ultimately impact empathy motivation. Disruptions to such value-based decision making with vmPFC lesions may make the rewards (or costs) integrated less into decisions to experience empathy. Therefore, examining how lesions to the vmPFC impact empathy motivation—in terms of decisions to approach or avoid it—and whether the perceptions of cognitive costs (e.g. effort and aversion) are associated with these decisions may clarify whether previously observed issues with empathy are due to motivation or ability.

The motivational precursors of empathy can be probed under certain study contexts (Keysers and Gazzola 2014). To offer an example of how this can help clarify empathy impairments, psychopathic offenders—often considered to have global empathy deficits—were studied under unconstrained contexts to examine whether they engage in empathy *spontaneously*. As a result, it was documented that these individuals did not exhibit *spontaneous* empathy under these testing conditions. However, they did exhibit empathy when it was encouraged, suggesting that they may not have deficits on empathy *ability* but rather may have deficits on (spontaneous) empathy *motivation* (Gillespie *et al.* 2014, Meffert *et al.* 2013). Adapting from similar frameworks, we sought to investigate whether the previously documented group differences in various types of empathy (e.g. emotional, cognitive) resulting from vmPFC lesions might be due to an unwillingness to deploy empathy—that is a reduction in empathy motivation.

### The current study

In the current study, we investigated the contribution of the vmPFC to empathy motivation using a lesion group comparison study. Lesion studies offer a clinically valuable and naturalistic opportunity to observe affective, behavioral, or cognitive differences in patients who have sustained focal brain damage and to test whether the damaged regions are *necessary* for different cognitive and affective functions. To our knowledge, no research to date has examined whether the altered empathic behavior of patients with vmPFC lesions may be due to differences in motivation to empathize.

To examine empathy motivation, we used a validated free-choice behavioral lab measure, the Empathy Selection Task (EST; Cameron *et al.* 2019). The EST provides participants with multiple choices to either approach or avoid empathy using an emotion-regulation strategy referred to as situation selection (Gross 2001), affording participants the opportunity to opt into situations in an *unconstrained* manner that either *encourage* or *discourage* empathy. We used two EST variants—one designed to target experience sharing/affective empathy and the other to target perspective taking/cognitive empathy. We opted to use both variants because they elicit separate facets of empathy (affective, cognitive) and provide different response modes to facilitate engagement with the task (i.e. written responses about emotional experiences, binary selection of positive/negative experiences). The EST can also reliably quantify individual differences on empathy motivation (Ferguson and Inzlicht 2023) and is associated with well-established multifaceted measures of self-reported dispositional empathy (Cameron *et al.* 2019, Scheffer *et al.* 2022, Ferguson and Inzlicht 2023).

We pre-registered our methods, hypothesis, and analysis plan during data collection (https://osf.io/rzva5). However, to simplify and improve the clarity of our results, we focus in this study on patients with vmPFC lesions. Patients with vmPFC lesions were compared to two other groups: (1) patients with brain damage outside of the vmPFC (brain-damaged comparison, BDC) and (2) demographically matched healthy comparison participants (HC). Our central focus was on comparing the patients with vmPFC lesions against BDC and HC groups (noted from here onward as “comparison groups”) rather than all originally recruited groups of a larger project which also included a fourth group of patients with amygdala lesions (Lakens 2024, Willroth and Atherton 2024). In a different manuscript, we reported on our findings for a sample of patients with amygdala lesions and their separately matched comparison groups (Scheffer *et al.* 2026) In this cited research, we found no evidence that patients with amygdala lesions show impairments on empathy motivation compared to brain-damaged and healthy comparison participants (see Supplementary Table 1).

We hypothesized that patients with vmPFC lesions would display lower empathy motivation—choosing the empathy decks less—compared to the other groups (BDC, HC) that would highlight motivation as a contributing factor to their previously documented deficits in empathy (H1). We also hypothesized that patients with vmPFC lesions would perceive more cognitive cost associated with empathy compared to the comparison groups (H2), given their documented impairments in perspective taking and emotion identification. As such, patients with vmPFC lesions were predicted to choose empathy less (Kool *et al.* 2010, Westbrook and Braver 2015, Westbrook *et al.* 2019). Third, we hypothesized that, across participants, perceiving empathy as more cognitively costly would be associated with choosing it less (H3) as documented in numerous previous studies using the EST (Cameron *et al.* 2019, Ferguson *et al.* 2020, Scheffer *et al.* 2022). Finally (reported in the Supplemental Materials), we examined the extent of task engagement on the affective EST (i.e. emotion word usage on empathy and non-empathy decks) and cognitive EST (i.e. attributions of positive and negative reactions from viewing images for the self and other trials) to ensure that variation in these did not unduly affect the main findings. We also report correlations of the proportion of vmPFC damage with proportion of choosing empathy in the affective and cognitive EST, and the correlations of individual differences (IRI, BIS-BAS, Self-Reported Altruism) with the proportion of choosing empathy in the affective and cognitive EST broken down by participant groups (see Supplemental Materials).

## Materials and methods

### Participants

We recruited 13 patients with focal unilateral or bilateral vmPFC lesions (3 left, 4 right, 6 bilateral), 13 patients with lesions outside of the vmPFC and other empathy-related regions (e.g. amygdala, insula; BDCs; 1 left, 8 right, 4 bilateral), and 16-demographically matched HC participants.

Demographic information is presented in Table 1. All patients had detailed neuropsychological testing and structural neuroimaging performed in the chronic epoch (>3 months post-lesion). Patients were screened to exclude individuals with a history of learning disabilities, psychiatric disorders, substance abuse, premorbid personality disorders, developmental epilepsy, and other neurological conditions not related to their focal lesion (Keifer and Tranel 2013). Participants were excluded using pairwise deletion from any analyses within a given EST (affective, cognitive) if they were missing at least one choice response for a deck in that given task.

**Table 1:** Participant demographics.

Participant group	Age *M* (*SD*)	Sex	Years of education *M* (*SD*)	Laterality	Chronicity years *M* (*SD*)
vmPFC Lesions	58.97 (15.30)	8 Male, 5 Female	14.54 (1.81)	3 Left 4 Right 6 Bilateral	24.01 (14.34)
BD comparison	63.21 (15.89)	9 Male, 4 Female	15.15 (2.94)	1 Left 8 Right 4 Bilateral	10.92 (7.39)
Healthy comparison	65.58 (9.90)	10 Male, 6 Female	16.50 (1.86)	NA	NA
Group tests	*F*(2, 39) = 0.84, *p* =* .*437, η_p_^2^ = 0.04	**χ** ^2^ = 0.20, *p* =* .*903	*F*(2, 39) = 2.96, *p* =* .*064, η_p_^2^ = 0.13	**χ** ^2^ = 2.73, *p* =* .*255	*t*(24) = 2.93, *p* = .007, 95% CI [3.86, 22.33]

*Note.* Case-matching was prioritized for age, then sex, then years of education for the vmPFC lesion group with individuals in the brain-damaged comparison and healthy comparison groups.

In the affective EST, we had to exclude one BDC patient for missing choice response data. In the cognitive EST, we had to exclude one vmPFC lesion patient and one BDC patient for missing choice response data. We also observed that two vmPFC lesion patients (one trial), two BDC patients (one trial), and one HC participant (two trials) had missing valence ratings, but we opted to retain their task data given this was minimal missing data (≤ 5%). All procedures were approved by the Institutional Review Board at the University of Iowa and are in accordance with the Declaration of Helsinki.

### Neuroanatomical analysis

Lesion boundaries were segmented manually for all patients’ scans according to standard procedures (Abel *et al.* 2016, Reber *et al.* 2021). Prior to 2006, lesion volumes were traced directly onto a template brain using the subjects’ scans using Brainvox and MAP-3 techniques (Frank et al. 1997). Lesions traced since 2006 were manually traced directly on the native T1-weighted scans using FSL (Smith et al. 2004) and then transformed to MNI152 space. A neurologist blinded to cognitive data and the study hypotheses reviewed and corrected the anatomical accuracy of each lesion mask in both native and MNI space (see Fig. 1).

**Figure 1:**
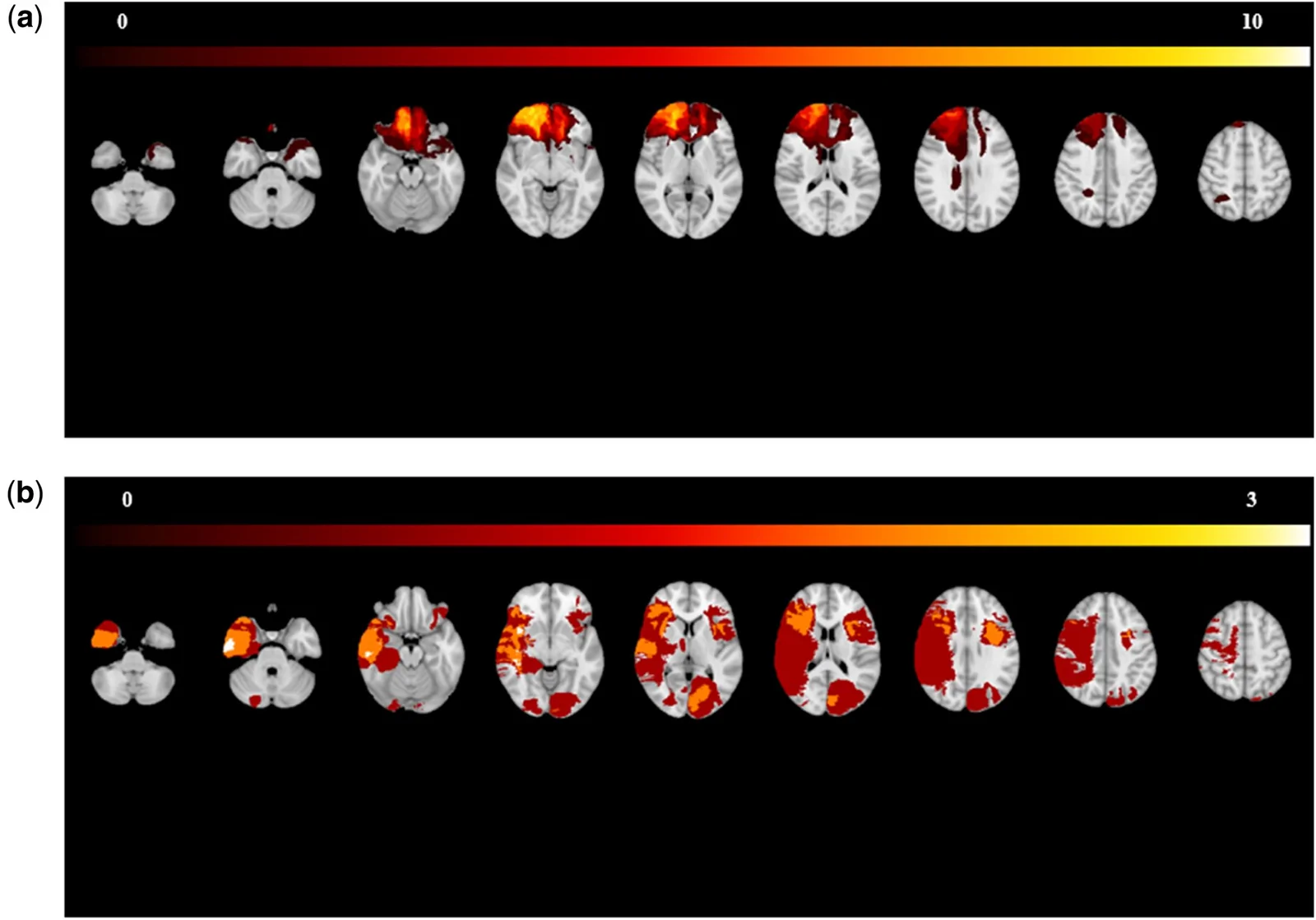
Lesion overlap map of patients with (a) vmPFC lesions and (b) brain-damaged comparisons. Spectrum of colors reflects greater overlap between lesion patients (yellow) compared to lesser overlap (red).

### Procedure

Participants completed a set of tasks that were programmed into an online Qualtrics survey. The researchers provided participants with a laptop computer with a keyboard and mouse that was used to input responses. Patients who were unable to type comfortably (due to hemiparesis, etc.) were allowed to respond verbally to the free response prompts, and their responses were typed verbatim by the experimenter (two patients with vmPFC lesions, two BDC participants). We retained these participants to maximize the inclusion of our sample given their choice data was complete. These participants exhibited choice proportion patterns that were in the range of their participant group average. We report the average choice patterns of the two individuals per group here and refer to Table 2 for the group patterns: ***affective EST***: *M*_vmPFC_ = 0.49, *M*_BDC_ = 0.30; ***cognitive EST***: *M*_vmPFC_ = 0.39, *M*_BDC_ = 0.18.

**Table 2:** Empathy-inducing deck choice proportion in the ESTs.

Participant group	Empathy choice *M* (*SD*)	95% CI of the *M*_diff_	*t*	*P*	*N*	Cohen’s *d*
**Affective EST**						
vmPFC lesions	0.47 (0.22)	[−0.17, 0.10]	−0.50	.626	13	−0.14
BD comparison	0.44 (0.17)	[−0.17, 0.05]	−1.14	.280	12	−0.33
Healthy comparison	0.37 (0.25)	[−0.26, 0.01]	−2.00	.064	16	−0.50
**Cognitive EST**						
vmPFC lesions	0.33 (0.23)	[−0.32, −0.02]	−2.56	.027	12	−0.74
BD comparison	0.33 (0.20)	[−0.30, −0.05]	−2.98	.012	12	−0.86
Healthy comparison	0.31 (0.21)	[−0.30, −0.07]	−3.48	.003	16	−0.87

*Note*: We had to exclude one BD comparison participant in the Affective EST and one vmPFC lesion and one BD comparison participant in the Cognitive EST.

#### Affective EST

Participants completed 40 trials of an affective variant of the EST to assess motivation to share in the experiences of other people (Cameron *et al.* 2019). Participants chose between a blue-colored “FEEL” deck (empathy choice) which instructed participants to focus on internal experiences and feelings of the person in the image, or a red-colored “DESCRIBE” deck (non-empathy choice) which instructed participants to focus on external features and appearances of the person in the image. After making a choice on a trial, they were shown one of 40 randomly selected images of a child refugee with instructions from their chosen deck. Participants could not submit their written response until at least 10 seconds had elapsed (see Fig. 2a).

**Figure 2:**
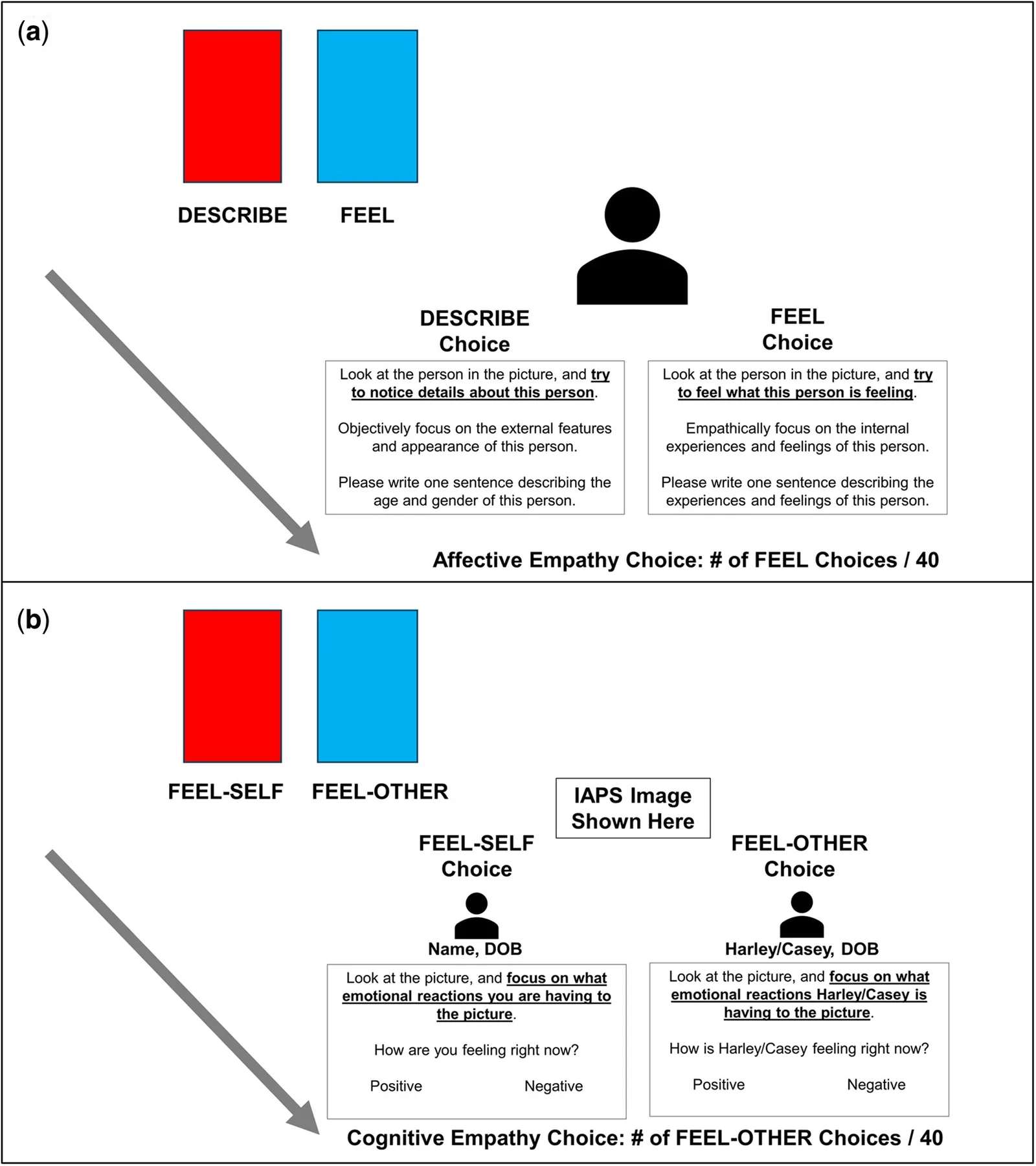
Affective (a) and cognitive (b) EST schematics. In the cognitive EST (b), Name, DOB on the FEEL-SELF deck refers to the participant’s piped-in information.

#### Cognitive EST

Participants completed 40 trials of a cognitive variant of the EST to assess motivation to imagine the emotional responses of another person (Cameron *et al.* 2019). Participants chose between a blue-colored “FEEL-OTHER” (empathy choice) which instructed them to concentrate on the emotional reaction that the other person was having, or a red-colored “FEEL-SELF” (non-empathy choice) which instructed them to concentrate on the emotional reaction that they themselves were having. Before participants began the task, they entered their first name as well as the date and month of their birth (e.g. “Larry, October 30”). Participants were also randomly assigned to either be paired with either Harley (October 3) or Casey (January 14) as the Feel-Other deck participant. On each trial, after participants made their choice, a randomly selected medium-arousal IAPS image was displayed and participants reported on whether they themselves (FEEL-SELF choice) were feeling positive or negative, or whether the other person (FEEL-OTHER choice) was feeling positive or negative. Participants could not submit their response until at least 10 seconds had elapsed (see Fig. 2b).

#### Post-task questionnaires

Participants provided responses to the following questions: “What was it like performing the task?”, “Did you develop a preference for one of the decks?,” and “How did you choose between the decks?” (Kool *et al.* 2010).

#### NASA Task Load Index

Participants answered the following NASA Task Load (Hart and Staveland 1988) Index questions in response to both decks within each task: “How mentally demanding was this deck?”, “How hard did you have to work to accomplish your level of performance with this deck?,” “How insecure, discouraged, irritated, stressed, and annoyed were you by this deck?,” and “How successful were you in accomplishing what you were asked to do in this deck?” The first two questions were combined to indicate perceived effort, the third question indicated aversiveness, and the fourth indicated perceived efficacy/success for engaging and achieving the deck’s instructions (Cameron *et al.* 2019, Westbrook *et al.* 2013).

#### Individual differences

After completing the aforementioned tasks, participants completed individual difference measures: Demographics (e.g. sex, education, and age), the Interpersonal Reactivity Index (Davis 1983) to assess participant endorsement of facets of empathy, the Behavioral Inhibition and Behavioral Activation System Scales (Carver and White 1994) to assess approach or avoidance tendencies in participants, and the Self-Reported Altruism Scale to assess prevalence of completing acts of other-oriented prosocial behaviors that ranged in effort (Rushton *et al.* 1981, see Supplemental Tables 5, 6, and 7).

### Analytic approach

#### Empathy motivation

To assess empathy motivation, we examined the proportion of choosing empathy: number of FEEL choices/40 total choices in the affective EST; and number of FEEL-OTHER choices/40 total choices in the cognitive EST. We report null-hypothesis significance test (NHST) analysis of variance (ANOVA) followed by confirmatory and separate Bayesian ANOVAs to determine the magnitude of the group differences effect size, 95% confidence region, and report the region of practical equivalence (ROPE) using the bayesanova package in R (i.e. whether the magnitude of the effect size was no effect, small, medium, or large and the highest percentage of support for the effect size; Kelter 2022a, 2022b).

#### Empathy’s cognitive costs

To assess perceptions of cognitive costs with empathy and non-empathy decks, we examined the ratings on the NASA Task Load Index (Hart and Staveland 1988) for effort, aversiveness, and efficacy for each deck collapsed across the three groups using NHST (see Tables 3 and 4). We examined the effect of deck (empathy, non-empathy) and the group-by-deck interactions. We also adopt Bayesian comparisons for confirmatory analyses to provide evidence toward the null or alternative hypothesis (see Table 5).

**Table 3:** Affective EST deck ratings on the NASA Task Load Index.

Participant group	Empathy deck *M* (*SD*)	Objective deck *M* (*SD*)	*M*_diff_ [95% CI]	*P*	**η**_p_^2^	Choice *r*	Choice *p*	*N*
**Effort**								
vmPFC lesions	2.88 (1.45)	2.46 (1.36)	[−0.14, 0.99]	.128	0.18	0.48	.100	13
BD comparison	3.08 (1.38)	2.96 (1.12)	[−0.44, 0.69]	.633	0.02	0.41	.184	12
Healthy comparison	3.44 (1.08)	2.28 (1.13)	[0.65, 1.66]	<* .*001	0.62	0.08	.765	16
**Aversion**								
vmPFC lesions	2.85 (1.52)	2.92 (1.50)	[−0.38, 0.22]	.584	0.03	−0.21	.484	13
BD comparison	2.83 (1.11)	2.67 (1.67)	[−0.88, 1.21]	.732	0.01	0.08	.794	12
Healthy comparison	3.19 (1.42)	2.25 (1.29)	[0.25, 1.62]	.011	0.36	−0.07	.792	16
**Efficacy**								
vmPFC lesions	3.85 (0.99)	3.46 (1.05)	[−0.14, 0.91]	.137	0.17	0.17	.569	13
BD comparison	3.83 (0.94)	4.08 (0.79)	[−0.86, 0.36]	.389	0.07	−0.13	.679	12
Healthy comparison	3.25 (1.13)	3.75 (1.00)	[−1.41, 0.41]	.261	0.08	0.05	.849	16

**Table 4:** Cognitive EST deck ratings on the NASA Task Load Index.

Participant group	Feel-other deck *M* (*SD*)	Feel-self deck *M* (*SD*)	*M*_diff_ [95% CI]	*P*	**η**_p_^2^	Choice *r*	*Choice p*	*N*
**Effort**								
vmPFC lesions	2.38 (1.09)	1.80 (1.03)	[−0.08, 1.24]	.078	0.26	−0.17	.594	12
BD comparison	1.92 (1.08)	1.58 (0.79)	[−0.29, 0.96]	.266	0.11	0.05	.868	12
Healthy comparison	2.34 (1.43)	1.47 (0.87)	[0.24, 1.51]	.010	0.37	−0.13	.622	16
**Aversion**								
vmPFC lesions	2.42 (1.31)	1.50 (1.17)	[0.04, 1.79]	.042	0.33	−0.55	.062	12
BD comparison	1.92 (1.24)	1.33 (0.65)	[−0.16, 1.32]	.111	0.21	−0.19	.553	12
Healthy comparison	1.69 (0.87)	1.25 (0.58)	[0.05, 0.83]	.029	0.28	0.27	.314	16
**Efficacy**								
vmPFC lesions	3.25 (1.06)	4.08 (1.16)	[−1.80, 0.14]	.085	0.25	−0.28	.369	12
BD comparison	4.00 (1.13)	4.33 (0.98)	[−0.90, 0.23]	.220	0.13	−0.20	.533	12
Healthy comparison	3.69 (1.40)	4.31 (1.08)	[−1.27, 0.02]	.055	0.22	0.40	.125	16

**Table 5:** Bayesian comparisons for cognitive costs by deck in the affective and cognitive EST.

Affective EST									
	Effort			Aversion			Efficacy		
Feel	$\bar{x}$	95% CI	ROPE	$\bar{x}$	95% CI	ROPE	$\bar{x}$	95% CI	ROPE
vmPFC									
vs. BDC	−0.17	[−0.58, 0.26]	54.03% none	0.01	[−0.39, 0.40]	76.56% none	0.01	[−0.26, 0.29]	90.78% none
vs. HC	−0.49	[−0.83, −0.11]	48.70% medium	−0.29	[−0.66, 0.09]	62.06% small	0.56	[0.28, 0.81]	70.48% medium

	**Effort**			**Aversion**			**Efficacy**		
			
**Describe**	$\bar{x}$	**95% CI**	**ROPE**	$\bar{x}$	**95% CI**	**ROPE**	$\bar{x}$	**95% CI**	**ROPE**

vmPFC									
vs. BDC	−0.39	[−0.68, −0.05]	66.58% small	0.17	[−0.23, 0.53]	55.93% none	−0.60	[−0.87, −0.27]	72.1% medium
vs. HC	0.15	[−0.14, 0.42]	66.01% none	0.49	[0.14, 0.80]	49.63% medium	−0.26	[−0.49, −0.01]	72.29% small

Cognitive EST									
	Effort			Aversion			Efficacy		
Feel-other	$\bar{x}$	95% CI	ROPE	$\bar{x}$	95% CI	ROPE	$\bar{x}$	95% CI	ROPE
vmPFC									
vs. BDC	0.43	[0.09, 0.73]	62.54% small	0.44	[0.03, 0.81]	54.58% small	−0.70	[−1.01, −0.34]	64.79% medium
vs. HC	0.03	[−0.29, 0.34]	83.51% none	0.69	[0.33, 1.00]	66.44% medium	−0.39	[−0.69, −0.06]	69.78% small

	**Effort**			**Aversion**			**Efficacy**		
			
**Feel-self**	$\bar{x}$	**95% CI**	**ROPE**	$\bar{x}$	**95% CI**	**ROPE**	$\bar{x}$	**95% CI**	**ROPE**

vmPFC									
vs. BDC	0.21	[−0.07, 0.46]	53.21% small	0.16	[−0.13, 0.42]	60.79% none	−0.22	[−0.50, 0.08]	57.28% small
vs. HC	0.32	[0.06, 0.56]	81.28% small	0.26	[−0.03, 0.53]	68.73% small	0.20	[−0.45, 0.07]	50.05% none

## Results

### Empathy motivation—proportion of empathy choice in the affective and cognitive EST

Across the entire sample, participants avoided the empathy deck in the affective EST (FEEL choice: *M *= 0.42, *SD *= 0.22, *t*(40) = −2.19, *P* = .034, 95% CI of the *M*_diff_ [−0.15, −0.01]). In the cognitive EST, participants avoided the empathy deck (FEEL-OTHER choice: *M *= 0.32, *SD *= 0.21, *t*(39) = −5.35, *P* < .001, 95% CI of the *M*_diff_ [−0.24, −0.11]).

Based on NHST, we did not find support of a group effect on choosing empathy for H1 (patients with focal vmPFC lesions will choose empathy less compared to comparison groups) in the affective EST (*F*(2, 38) = 0.73, *P* = .488, η_p_^2^ = 0.04) or in the cognitive EST (*F*(2, 37) = 0.02, *P* = .979, η_p_^2^ = 0.00). Among the two ESTs, we observed a moderate correlation between the proportion of choosing empathy in the affective and cognitive EST in the full sample (*r *= 0.39, *P* = .012, 95% CI [0.09, 0.62]), which suggests that performance on these tasks may be related (see Fig. 3, Table 2).

**Figure 3:**
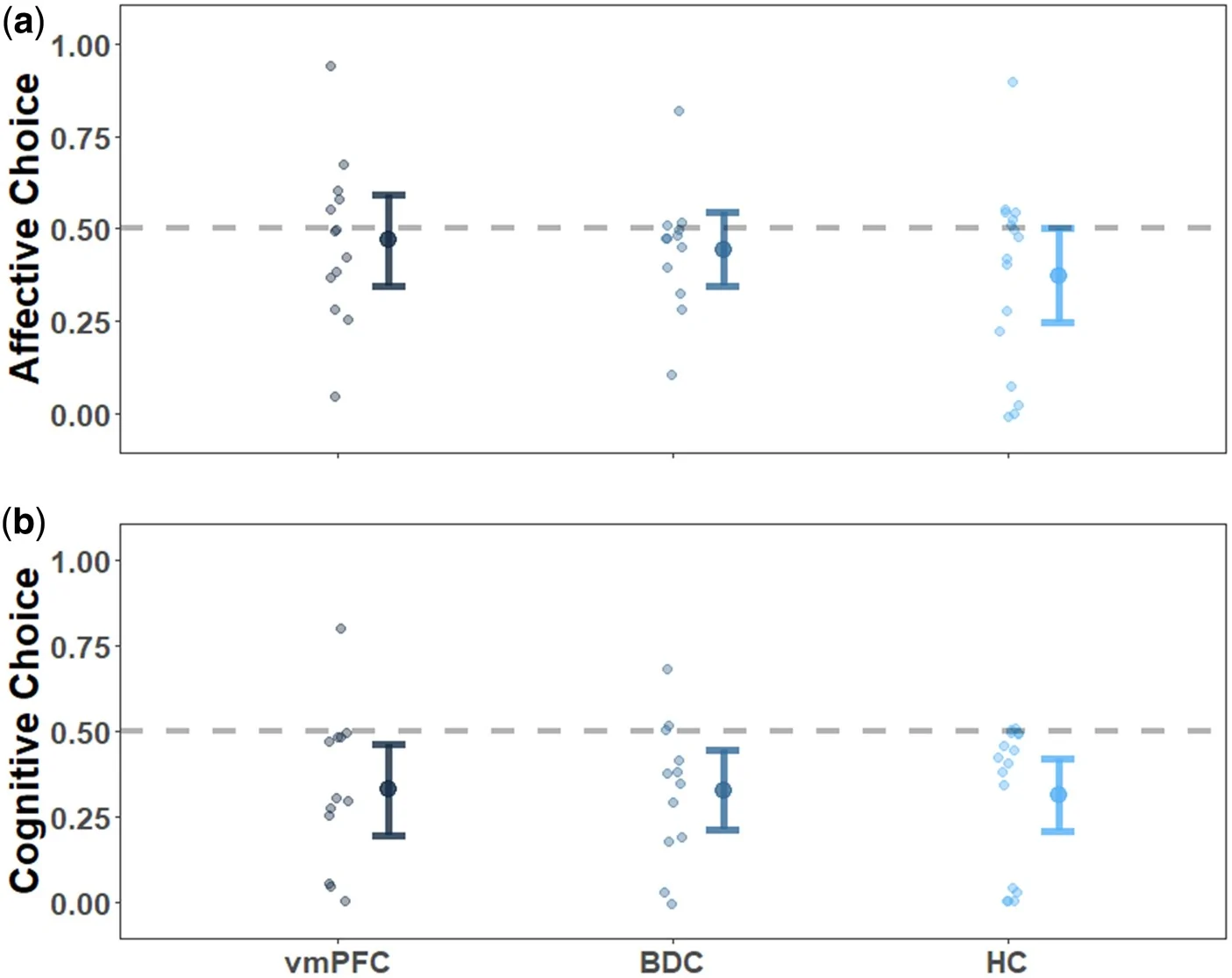
Choice proportion in the affective EST (a) and cognitive EST (b). BDC refers to the brain-damaged comparison group, and HC refers to the healthy comparison group. Dashed grey line reflects chance proportion (.50). Individual participant scores are jittered slightly in position to prevent overlap around the group means (black dots) and 95% confidence intervals (error bars).

### Bayesian comparisons confirm no group effects on empathy motivation

For the affective EST no group effect was observed between patients with vmPFC lesions and BDC patients (posterior mean = 0.04, 95% CI [−0.05, 0.12]; 100% posterior probability inside ROPE of no effect) or between patients with vmPFC lesions and HC participants (posterior mean = 0.14, 95% CI [0.06, 0.21]; 98.65% posterior probability inside ROPE of no effect) in their proportion of choosing empathy.

For the cognitive EST, there were violations of normality with the preferences for avoiding empathy. Therefore, we report and interpret these Bayesian comparisons with caution. No Group effect was observed between patients with vmPFC lesions versus BDC patients (posterior mean = 0.00, 95% CI [−0.09, 0.10]; 100% posterior probability inside ROPE of no effect) or between patients with vmPFC lesions versus HC participants (posterior mean = 0.02, 95% CI [−0.06, 0.10]; 100% probability inside ROPE of no effect).

Altogether, the results do not provide support for the hypothesis that patients with vmPFC lesions differ from the comparison groups on empathy choice proportion (see Fig. 3, Table 2). On the contrary, the findings can be taken to support the conclusion that patients with vmPFC lesions do not exhibit remarkable differences in their motivation to engage in affective and cognitive empathy, compared to patients with brain damage outside the vmPFC and participants with no brain damage.

### Empathy’s cognitive costs—NASA Task Load Index ratings

In the affective EST, there was a deck effect, such that participants reported the empathy deck more effortful (*F*(1, 38) = 15.32, *P* < .001, η_p_^2^ = 0.29), more aversive (*F*(1, 38) = 3.13, *P* = .085, η_p_^2^ = 0.08) than the non-empathy deck, but there was no Deck effect on efficacy for the empathy deck compared to the non-empathy deck (*F*(1, 38) = 0.36, *P* = .552, η_p_^2^ = 0.01). In the cognitive EST, there was a deck effect, such that participants reported the empathy deck more effortful (*F*(1, 37) = 11.82, *P* = .001, η_p_^2^ = 0.24), more aversive (*F*(1, 37) = 13.85, *P* < .001, η_p_^2^ = 0.27), and reported less efficacy (*F*(1, 37) = 9.24, *P* = .004, η_p_^2^ = 0.20). Therefore, we replicated earlier research, which found that participants generally rated the empathy decks more cognitively costly compared to the non-empathy decks within each of the two ESTs (affective, cognitive; Cameron *et al.* 2019). However, we did not find consistent support for H2 with no support for a group-by-deck effect (compared to the comparison groups, patients with focal vmPFC lesions ratings of cognitive costs would be greater for the empathy deck compared to the non-empathy deck in the two ESTs; see Fig. 4, Tables 3 and 4).

**Figure 4:**
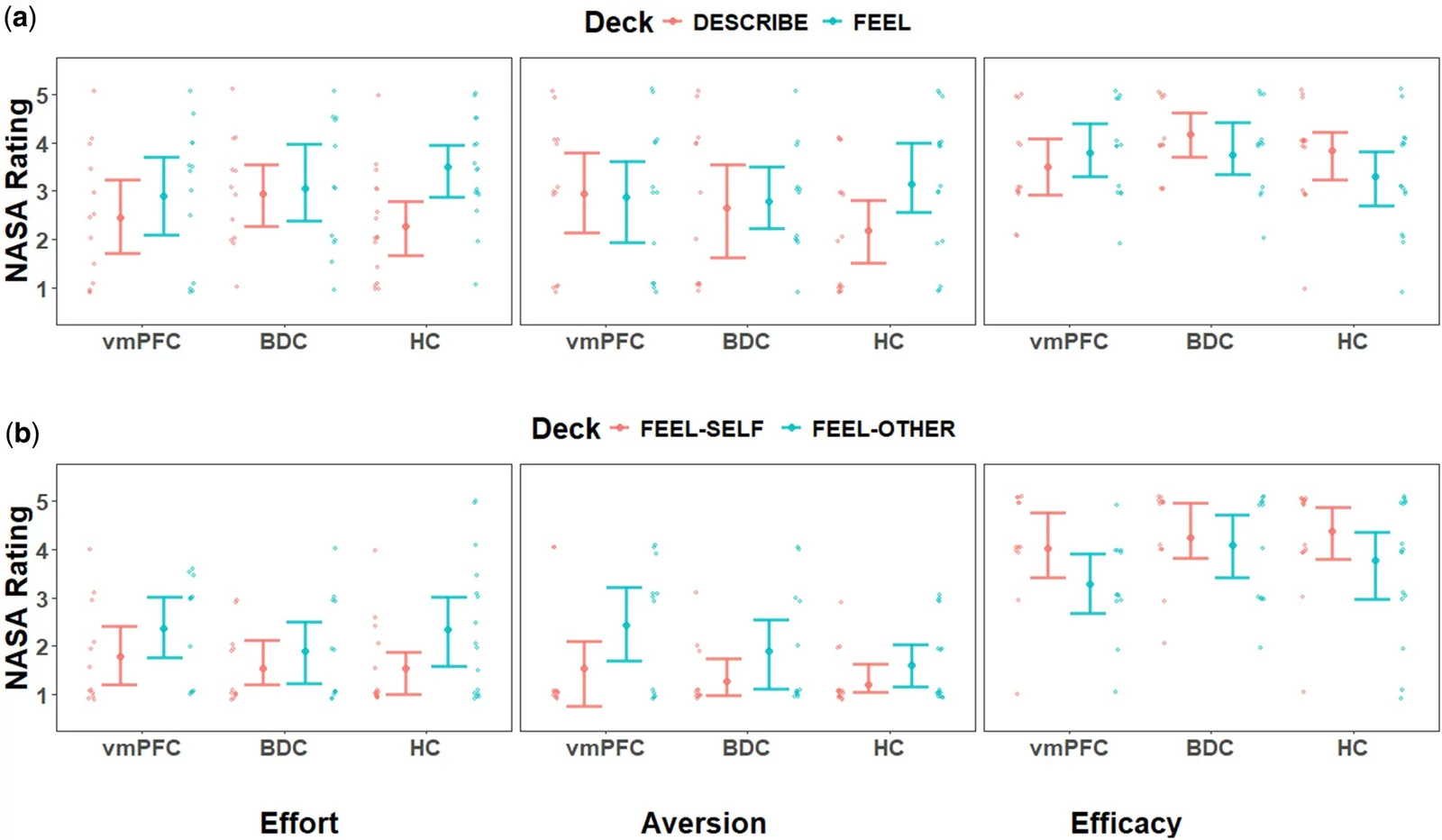
Self-reports of cognitive costs in the (a) affective and (b) cognitive EST. BDC refers to the brain-damaged comparison group, and HC refers to the healthy comparison group. Individual participant scores are jittered slightly in position to prevent overlap beside the group means (larger dots) and 95% confidence intervals (error bars).

### Empathy felt less effortful to patients with vmPFC lesions

In the affective EST, there was a significant group-by-deck interaction on effort (*F*(2, 38) = 4.73, *P* = .015, η_p_^2^ = 0.20), but not on aversion (*F*(2, 38) = 2.72, *P* = .079, η_p_^2^ = 0.13) or on efficacy (*F*(2, 38) = 1.74, *P* = .189, η_p_^2^ = 0.08).

In probing the interaction within each participant group, patients with vmPFC lesions did not exhibit evidence of a deck effect, with no evidence supporting perceiving differences between the empathy and non-empathy deck (*t*(12) = 1.64, *P* = .128) and neither did BDC participants (*t*(15) = 0.49, *P* = .633), but HC participants (*t*(15) = 4.90, *P* < .001) exhibited evidence of a deck effect as participants reported the empathy deck more effortful than the non-empathy deck.

In the cognitive EST, there were no significant group-by-deck interactions on effort (*F*(2, 37) = 0.86, *P* = .431, η_p_^2^ = 0.04), on aversion (*F*(2, 37) = 0.68, *P* = .514, η_p_^2^ = 0.04), or on efficacy (*F*(2, 37) = 0.50, *P* = .610, η_p_^2^ = 0.03).

Confirmatory Bayesian ANOVAs are covered more elaborately in Table 5. Here, we summarize the group effects on cognitive costs for the empathy deck. In the affective EST, for effort, comparisons revealed a very small effect that patients with vmPFC lesions rated empathy less effortful than BDC patients (posterior mean = −0.17, 95% CI [−0.58, 0.26], 54.03% posterior probability within ROPE of no effect) and a medium effect against HC participants (posterior mean = −0.49, 95% CI [−0.83, −0.11], 48.70% posterior probability within ROPE of medium effect). For aversion, Bayesian comparisons revealed no effect against BDC patients (posterior mean = 0.01, 95% CI [−0.39, 0.40], 76.56% posterior probability within ROPE of no effect) and a small effect against HC participants (posterior mean = −0.29, 95% CI [−0.66, 0.09], 62.06% posterior probability within ROPE of small effect). For efficacy, Bayesian comparisons revealed no effect when comparing patients with vmPFC lesions against BDC patients (posterior mean = 0.01, 95% CI [−0.26, 0.29], 90.78% posterior probability within ROPE of no effect) and a medium effect against HC participants (posterior mean = 0.56, 95% CI [0.28, 0.81], 70.48% posterior probability within ROPE of medium effect). In summary, patients with vmPFC lesions rated the affective EST’s empathy deck as less effortful than comparison groups (see Table 5).

For the cognitive EST, when comparing patients with vmPFC lesions against BDC patients or HC participants, Bayesian comparisons revealed across effort and efficacy that more evidence supported no effects. However, patients with vmPFC lesions rated the empathy deck more aversive than comparison groups (against BDC patients: posterior mean = 0.44, 95% CI [0.03, 0.81], 54.58% posterior probability within ROPE of small effect; against HC participants: posterior mean = 0.69, 95% CI [0.33, 1.00], 66.44% posterior probability within ROPE of medium effect). In summary, for cognitive empathy, there were no consistent group differences across NHST and Bayesian analyses, although Bayesian analyses revealed that patients with vmPFC lesions reported cognitive empathy more aversive than comparison groups (see Table 5).

### Cognitive costs did not correlate with empathy motivation

We did not find consistent support for H3 (higher ratings of effort, aversiveness, and efficacy on the empathy decks compared to the non-empathy decks within each task will be associated with the proportion of choosing empathy). Difference scores were computed by subtracting the NASA Task Load Index rating for the non-empathy deck from the empathy deck to create scores on effort, aversiveness, and efficacy (e.g. Effort_FEEL_ − Effort_DESCRIBE_ = Effort_diff_). We examined whether these difference scores separately were associated with the proportion of choosing empathy within each of the two ESTs (affective, cognitive). In the affective EST, there were no significant associations between the proportion of choosing empathy and reporting it more effortful (*r *= 0.17, *P* = .294, 95% CI [−0.15, 0.45]), aversive (*r *= −0.10, *P* = .531, 95% CI [−0.40, 0.21]), or efficacious (*r *= 0.09, *P* = .566, 95% CI [−0.22, 0.39]) than the non-empathy deck. In the cognitive EST, there was no associations with the proportion of choosing empathy and rating it as more effortful (*r *= −0.10, *P* = .534, 95% CI [−0.40, .22]), aversive (*r *= −0.20, *P* = .217, 95% CI [−0.48, .12]), or efficacious (*r *= 0.00, *P* = .980, 95% CI [−0.31, .32]) than the non-empathy deck (see Tables 3 and 4). We reported comparisons of the correlations between cognitive costs with empathy and proportion of choosing empathy (empathy motivation) in the two ESTs in Supplemental Materials, finding no consistent evidence of group differences. However, it is important to note that our sample size is under-powered to detect individual difference correlations (i.e. for effect size *f* = .25 [medium] for a two-tailed correlation at 80% power and .05 alpha level, we would require 120 participants; Faul *et al.* 2009).

## Discussion

On two different free-choice tasks examining motivation to engage in affective and cognitive empathy, patients with vmPFC lesions did not exhibit abnormal empathy motivation by selecting empathy less than comparison groups. Additionally, the more affective components of empathy—sharing and identifying emotional experiences—were reported to be less effortful, while the cognitive components—imagining the emotional experiences of other people—were reported to be more aversive when compared to the brain-damaged and HC groups (but this latter finding was only evident using Bayesian analyses). Earlier research has highlighted that the vmPFC is involved in theory of mind and empathy (Shamay-Tsoory *et al.* 2006, Leopold *et al.* 2012), and it is possible that damage to these regions disrupts the necessary emotion recognition mechanisms that make this process feel more effortful and/or less aversive.

At face value, our findings can be taken to indicate that patients with vmPFC damage have preserved empathy motivation, despite their real-world impairments in empathy accuracy and showing concern for the well-being of others. In the current research, we did not document whether these specific patients with vmPFC lesions had deficits in empathic ability. Further, on our measure of empathy motivation, these patients did not exhibit differences with engaging with empathy based on their performance on the empathy decks in the ESTs (see Supplemental Material). Nevertheless, the distinction of preserved empathy motivation in the face of real-world impairments in empathy ability may be apparent when the deployment of empathy in contexts where a social partner is suffering (e.g. crying in distress) results in empathic engagement but inappropriate responding due to issues of recognizing the partner’s emotional experience (e.g. sadness) or absence of concern for their feelings. Moreover, patients with vmPFC lesions are known to perform well in laboratory contexts but may be unable to deploy empathy naturalistically when a situation calls for it. More research is needed to understand how patients with vmPFC lesions deploy empathy and exhibit prosocial behavior with social interaction partners. The lack of group differences between patients with vmPFC lesions and comparison groups on empathy motivation may be in part due to the ratings of cognitive costs for the affective component (and its associated mental effort) and cognitive component (and its associated aversiveness) that were obtained in this research.

We have conceptualized the results of the EST as has been done previously (Cameron *et al.* 2019, Ferguson *et al.* 2020, Scheffer *et al.* 2022, 2026). In the current study, we isolate motivation to approach empathy (i.e. choosing empathy decks compared to non-empathy decks more) and do not focus on the experience of empathy or its ability. Motivation to empathize and ability to empathize may have different impacts on interpersonal interactions (e.g. someone who *tries* to empathize but cannot empathize due to issues of ability may be treated differently than someone who *does not try* to empathize but has the ability to empathize).

Of course, empathy measured through a lab paradigm may not always predict empathy outside of the lab. Therefore, future research can investigate whether empathy motivation in these paradigms necessarily links with opting into empathy in the real-world similarly applies (e.g. investigating applications of empathy, such as engaging with the emotional suffering of another person or in a conversation with a loved one; Depow *et al.* 2021). It has not escaped our attention that the current results were obtained in a structured laboratory paradigm, off-line, not in the “real world.” This leaves open the question of whether patients with vmPFC lesions would show normal empathy motivation and/or effectively deploy empathy to motivate helping behavior. There is a possibility that we might not observe this: similar to persons with psychopathy, patients with vmPFC lesions have classically been noted to “talk a good game” in structured, off-line laboratory settings, but then fail to deploy appropriate behavior—like exhibiting empathic concern and helping—in the real world (Blair 2007, Eslinger and Damasio 1985). The paired deficits that come with vmPFC damage may explain why more structured compared to unstructured real-world settings may provide issues for these individuals with decision-making and planning. To that point, research which uses less instructional, structured, and closed formats may better reveal the issues associated with vmPFC damage for real-world decision-making (Tranel *et al.* 2007).

Empathy motivation in the two ESTs could be conceptualized as either empathy approach or empathy avoidance due to the binary forced-choice nature of the task. Binary tasks have been used frequently in the domain of decision-making to study such concepts as mental effort avoidance (e.g. Kool *et al.* 2010, Westbrook *et al.* 2013) and empathy avoidance (Cameron *et al.* 2019, Scheffer *et al.* 2022). Independent trials which isolate approaching (accepting) or avoiding (rejecting) empathy decks that are orthogonal to approaching (accepting) or avoiding (rejecting) non-empathy decks could clarify which motivation is more representative of the findings in this research. In fact, future research could adapt the EST to provide separate trials that allow participants to accept or reject trials that propose completing the two separate decks.

This research supports the value of studying motivation-based deficits along with capacity-based deficits to discriminate the source of functional impairments (Keysers and Gazzola 2014, Zaki 2014, Cameron 2018) as both contribute to empathy’s success. Contributions of the vmPFC may be implicated with accurately identifying and recognizing the emotional experiences of others (Grattan *et al.* 1994, Leigh *et al.* 2013, Hillis 2014), as well as generating the physiological responses that link with emotion sharing and perception (Damasio *et al.* 1990). It may be the case that patients with vmPFC patients have similar levels of empathy motivation as others, but after empathizing, may not generate the same physiological reactions that increase concern to subsequently *help* others.

Previous work has suggested that psychopathic traits may be associated with a lack of motivation to empathize, but an intact ability to do so (Baskin-Sommers and Newman 2014). Our findings further indicate that patients with vmPFC lesions may exhibit no remarkable impairments in empathy motivation on these ESTs, but these patients might suffer from compromised accuracy in affective and/or cognitive empathy in other tasks, especially in complex social situations. Although these patterns of impairment might lead to similar behaviors, they differ fundamentally.

### Limitations

The generalizability of our findings is limited by our sample size, characteristics, and statistical power. A sensitivity analysis (Faul *et al.* 2007) of our investigation of empathy motivation using the lesion comparison method indicated that we would have the ability to assess differences for the patients with vmPFC lesions from comparison groups with an effect size *f* =* .*50 (medium). Nevertheless, group lesion studies have clinical value for studying changes in behavior (Adolphs 2016), are quite rare (McCurdy *et al.* 2022) given the scarcity of patients with focal lesions to these regions, and generally have larger effect sizes given the profound changes that occur with lesion damage (Bezeau and Graves 2001). We additionally supplement our primary NHST analyses with Bayesian frequentist comparisons to report whether we obtained evidence for the null hypothesis or against it.

Using behavioral measures such as the EST can help better isolate the experience of empathy, study multiple choices of opting into empathy, and probe why people may or may not want to experience it (Mook 1983). Accordingly, future research can attempt to investigate empathy motivation in patients with acquired vmPFC damage using more ambiguous and unconstrained contexts that can model the multiple subgoals that people balance with choosing to experience empathy (Zaki 2014, Cameron *et al.* 2022).

## Conclusion

Individuals with lesions to the vmPFC often exhibit impairments in empathy, characterized by deficits with identifying and recognizing the emotions of others and generating physiological responses that may facilitate emotion sharing. This has contributed to theories of the vmPFC that link its dysfunction to “psychopathy” and socioemotional impairments. Here, we found that individuals with vmPFC lesions did not display deficits in motivation to empathize with strangers in a free-choice lab paradigm. Although we did not observe an effect on empathy motivation here, our approach still suggests that it is important to test for motivation and not assume a capacity difference. More research is needed to understand the factors which contribute to empathy motivation and the extent to which motivation and capacity-based deficits with empathy contribute to empathic functioning in everyday life.

## Supplementary Material

nsag072_Supplementary_Data

## Data Availability

Data, syntax, and analytic code are available: https://osf.io/u8y7p/ .
