# Supplementary material for: Is damage to the ventromedial prefrontal cortex associated with impaired empathy? A neuropsychological study using an empathy motivation task
Source: Soc Cogn Affect Neurosci. 2026 Sep 2;21(1):nsag072. doi: 10.1093/scan/nsag072 (PMC13743529; doi:10.1093/scan/nsag072)
Supplement: Supplementary file 1 [file nsag072_supplementary_data.docx]

**Supplementary Material**

We hypothesized that patients with vmPFC lesions would have stronger correlations between perceiving empathy as cognitively costly and choosing it less than comparison groups (supplemental H4). We additionally report on the engagement of participant groups on the affective and cognitive EST to ensure that any differences with how these groups adhered to the deck instructions did not unduly contribute to any choice proportion findings. Exploratory analyses are also covered in Supplemental Material to examine the association between the proportion of vmPFC damage with choosing empathy (given prior research documenting the necessity of the vmPFC on empathy; Janowski *et al.*, 2013; Lockwood *et al.*, 2024). Where applicable, we used pairwise deletion methods to handle missing data.

**Comparisons of Empathy Choice Correlations with Cognitive Costs**

We did not obtain consistent support for Supplemental H4 (the vmPFC lesion group will have higher correlations between the proportion of choosing empathy and the relative perceived effortfulness, aversiveness, and efficaciousness of empathy compared to non-empathy deck within each EST). We conducted comparisons of the associations of cognitive costs and empathy choice (see Tables 3 and 4) using the cocor package shiny web app (Diedenhofen and Musch, 2015). The comparison test accounts for participant group sample size and correlation coefficients, and we conducted these at a .05 alpha level, 95% confidence interval, null value of 0, using two-tailed tests (see Supplemental Table 2). We only observed that in the cognitive EST, patients with vmPFC lesions had a weaker correlation between rating the empathy deck more aversive and choosing it less compared to the healthy comparison group.

**Affective EST Engagement**

Given the affective EST requests responses after participants make their deck selection (FEEL, DESCRIBE), we examined whether the content of their written responses differed by participant group. The affective EST presents the images of child refugees. Many of these children are expressing negatively-valenced emotions (e.g., sadness, fear), therefore, we coded whether participants wrote in more negative or positive emotion words within their responses, and broke this down by their participant group to determine whether any groups may have unexpectedly reported more or less negative and positive emotion words. In exploratory analyses, we conducted a series of ANOVAs to examine whether the use of positive or negative emotion words would vary based on deck choice (FEEL/empathy, DESCRIBE/non-empathy) and participant group. We would expect more emotion words on the empathy compared to non-empathy deck in this task, but we had no prior predictions about the participant groups varying in their emotion word usage.

***Revised Emotion Word Dictionary Creation***

We assigned a coding team—blinded to the participants’ deck choices (FEEL empathy; DESCRIBE non-empathy) as well as the participant groups—to review these written responses. In their review, they identified words that were incorrectly identified as emotion words in the context of the written text (e.g., “like”) along with emotion words that were not counted. We then created a combined emotion word dictionary that removed incorrectly identified emotion words in context, incorrectly unidentified emotion words, and a combination of the earlier two emotion word dictionaries (positive, negative). We used this dictionary to replicate earlier analyses to ensure that any findings were not due to unidentified emotion words or emotion words that were incorrectly identified and not representing an emotion or feeling state.

**Affective EST Engagement Results**

For use of positive emotion words (see Supplemental Table 3) participants overall were more likely to use a positive emotion word on the empathy compared to the non-empathy deck (b = 1.19, *SE*(b) = 0.42, *p* = .004), but there were no differences across participant groups (b = -0.24, *SE*(b) = 0.21, *p* = .266) or an interaction of deck choice and participant group (b = 0.06, *SE*(b) = 0.20, *p* = .770).

For use of negative emotion words (see Supplemental Table 3), participants overall were more likely to use a negative emotion word on the empathy compared to the non-empathy deck (b = 2.32, *SE*(b) = 0.37, *p* < .001), but there were no differences across participant groups (b = 0.14, *SE*(b) = 0.21, *p* = .511) or an interaction of deck choice and participant group (b = -0.17, *SE*(b) = 0.17, *p* = .310).^[1]^

***Revised Emotion Word Dictionary Results***

For use of the emotion words in this revised emotion word dictionary, participants overall were more likely to use an emotion word on the empathy compared to the non-empathy deck (b = 3.21, *SE*(b) = 0.65, *p* < .001), but there were no differences across participant groups (b = 0.10, *SE*(b) = 0.29, *p* = .727) and no interaction of deck choice and participant group (b = 0.00, *SE*(b) = 0.21, *p* = .991).

In summary, these results suggest that participants were more likely to use emotion words on empathy compared to non-empathy deck trials. However, there was no evidence suggesting that patients with vmPFC lesions completed the affective EST instructions differently from comparison groups, implying that they were as accurate as comparison groups in identifying the negative emotions of the individuals in the images.

**Cognitive EST Engagement**

We explored whether participants recognized that the other person would either feel more positive or more negative in responses to the emotional images. The images selected from the International Affective Picture System have normative valence ratings (Lang *et al.*, 1997), and in our set, 20 images were more positive and 20 images were more negative. Therefore, we examined how these valence ratings matched what participants identified in other people as well as in themselves based on the FEEL-OTHER and FEEL-SELF deck choices, respectively.

***Cognitive EST Engagement Results***

Participants in all groups appeared to accurately identify the valence of the images on this task. When choosing the empathy deck for the more positive images, there were no significant group differences in the proportion of rating the other person as feeling more positive (*F*(2, 30) = 0.77, *p* = .471, η_p_^2^ = 0.05). When choosing the empathy deck for more negative images, there again were no significant group differences in the proportion of rating the other person as feeling more negative (*F*(2, 29) = 1.14, *p* = .335, η_p_^2^ = 0.07).

When choosing the non-empathy deck for more positive images, there were no group differences in the proportion of reporting feeling more positive (*F*(2, 37) = 0.14, *p* = .869, η_p_^2^ = 0.01). When choosing the non-empathy deck for more negative images, again there were no group differences in the proportion of reporting feeling more negative (*F*(2, 37) = 1.80, *p* = .180, η_p_^2^ = 0.09).

In summary, the results do not imply that patients with vmPFC lesions completed the cognitive EST instructions differently from comparison groups.

**Exploratory Analyses**

***Proportion of vmPFC Damage and Empathy Motivation***

We examined how laterality (left, right) of damage to the vmPFC or vmPFC was associated with proportion of choosing empathy in the experience sharing and mentalizing ESTs. This provided another means of evaluating the contribution of these neural regions in motivation to empathize, by examining how the proportion of their loss is associated with empathy choice (Lashley, 1950; Baas *et al.*, 2004). In these analyses, we excluded the healthy comparison participants given they did not have brain damage to examine, as well as one vmPFC lesion and one brain-damaged comparison patient who did not have available mapped data. We did not find statistically significant associations with the proportion of left or right vmPFC damage with choosing the empathy deck in the affective or cognitive EST (see Supplemental Table 4).

**Individual Differences and Empathy Choice**

We observed no support for significant group differences on individual differences on the Interpersonal Reactivity Index (IRI), Behavioral Activation System (BAS) and Behavioral Inhibition System (BIS), and Self-Reported Altruism (see Supplemental Table 5). However, we observed in patients with vmPFC lesions that higher ratings on IRI-Fantasy and IRI-Empathic Concern were associated with higher proportion of choosing empathy in the affective EST. Additionally, higher ratings on Self-Reported Altruism were also associated with higher proportion of choosing empathy in the affective EST for patients with vmPFC lesions. For healthy comparison participants, higher ratings on IRI-Personal Distress were associated with a higher proportion of choosing empathy in the affective EST (see Supplemental Table 6). When comparing the magnitude of correlations of individual differences with choosing empathy in the ESTs, we observed that patients with vmPFC lesions had stronger magnitudes of these correlations in the affective EST and IRI-Perspective Taking when compared to brain-damaged and healthy comparison participants (see Supplemental Table 7).

**References**

Baas, D., Aleman, A., Kahn, R.S. (2004). Lateralization of amygdala activation: a systematic review of functional neuroimaging studies. *Brain Research Reviews*, **45**, 96–103

Diedenhofen, B., Musch, J. (2015). cocor: A Comprehensive Solution for the Statistical Comparison of Correlations J. Olivier (ed). *PLOS ONE*, **10**, e0121945

Janowski, V., Camerer, C., Rangel, A. (2013). Empathic choice involves vmPFC value signals that are modulated by social processing implemented in IPL. *Social Cognitive and Affective Neuroscience*, **8**, 201–8

Lang, P.J., Bradley, M.M., Cuthbert, B.N. (1997). International affective picture system (IAPS): Technical manual and affective ratings. *NIMH Center for the Study of Emotion and Attention*, **1**, 3

Lashley, K.S. (1950). In search of the engram. In Society for Experimental Biology, Physiological mechanisms in animal behavior. (Society's Symposium IV.) (pp. 454–482). Academic Press.

Lockwood, P.L., Cutler, J., Drew, D., et al. (2024). Human ventromedial prefrontal cortex is necessary for prosocial motivation. *Nat Hum Behav*, ***8***(7), 1403-1416.

**Supplemental Table 1**

*Pre-registration, Analytical, and Comparison Deviations*

| **Hypothesis** | **Original** | **Deviation** |
| --- | --- | --- |
| H1 | Neurological patients with focal amygdala lesions will choose empathy more compared to patients with focal vmPFC lesions, brain-damaged comparison patients, and demographically-matched healthy comparison participants. | Patients with vmPFC lesions will choose empathy less than the comparison groups.  [Comparisons for patients with amygdala lesions are reported in a separate manuscript] |
| H2 | Neurological patients with focal amygdala lesions will perceive less effort, aversion, and inefficacy with empathy, measured through the NASA Task Load Index, compared with patients who have focal vmPFC lesions, brain-damaged comparison patients, and demographically-matched healthy comparison participants. | Patients with vmPFC lesions will perceive more cognitive cost associated with empathy (relative to the contrast deck) compared to the comparison groups. |
| H3 | Choosing empathy in the Empathy Selection Task will correlate with perceiving lesser effort, aversion, and inefficacy with empathy as measured through the NASA Task Load Index. | ***No Deviation***. |
| H4  (or H3a) | Patients with focal amygdala lesions will have lower correlations of NASA Task Load Index effort, aversion, and inefficacy with empathy choice compared to patients with focal vmPFC lesions, brain-damaged comparison patients, and demographically-matched healthy comparison participants. | Patients with vmPFC lesions will have higher correlations of NASA Task Load Index effort, aversion, and inefficacy with empathy choice compared to brain-damaged comparison patients and demographically-matched healthy comparison participants. |
| Analysis Approach | Amygdala (*N* = 21), vmPFC (*N* = 13), brain-damaged comparison (*N* = 35), healthy comparison (*N* = 40) with ANOVAs on group-effects. | vmPFC (*N* = 13), brain-damaged comparison (*N* = 13/35), healthy comparison (*N* = 16/40) with ANOVAs on group-effects and Bayesian frequentist analyses. |
| Exclusions | Participants with blank responses on either of the two Empathy selection Tasks will be excluded from analyses of that particular Empathy Selection Task. | In the affective EST, we excluded one brain-damaged comparison patient for missing choice data. In the cognitive EST, we excluded one vmPFC lesion patient and one brain-damaged comparison patient for missing choice data. |

*Note*: Brain-damaged comparison and healthy comparison participants were matched with patients with vmPFC and amygdala lesion samples separately using the case-matching feature in SPSS (on age, sex, and education level).

**Supplemental Table 2**

*Comparisons of Correlation Magnitudes for Cognitive Cost Ratings with Empathy Choice*

|  | **vmPFC Lesion Group** | | | |
| --- | --- | --- | --- | --- |
|  | vs. BD Comparison | | vs. Healthy Comparison | |
| **Affective EST** | **Z** | ***p*** | **Z** | ***p*** |
| Effort | 0.19 | .849 | 1.05 | .293 |
| Aversiveness | -0.64 | .523 | -0.34 | .734 |
| Efficacy | 0.66 | .510 | 0.29 | .773 |
| **Cognitive EST** | **Z** | ***p*** | **Z** | ***p*** |
| Effort | -0.47 | .638 | -0.09 | .925 |
| Aversiveness | -0.90 | .366 | -2.06 | .039 |
| Efficacy | -0.18 | .857 | -1.64 | .101 |

**Supplemental Table 3**

*Affective EST Emotion Word Use Proportion*

|  | **vmPFC Lesion**  *M* (*SD*) | **BD Comparison**  *M* (*SD*) | **Healthy Comparison**  *M* (*SD*) |
| --- | --- | --- | --- |
| **FEEL**  Positive  Negative | 0.13 (0.10)  0.27 (0.15) | 0.12 (0.08)  0.20 (0.09) | 0.09 (0.08)  0.23 (0.15) |
| **DESCRIBE**  Positive  Negative | 0.04 (0.05)  0.07 (0.10) | 0.08 (0.13)  0.11 (0.11) | 0.04 (0.06)  0.13 (0.21) |

*Note*. Proportion of trials in which participants used a positive or negative emotion word out of the total number of trials (40).

**Supplemental Table 4**

*Proportion of Damage Associations with Empathy Choice Proportion*

|  | ***b*** | ***t*** | ***p*** | ***N*** |
| --- | --- | --- | --- | --- |
| **Affective EST** | | | | |
| L vmPFC | 0.15 | 0.64 | .528 | 23 |
| R vmPFC | -0.13 | -0.82 | .420 | 23 |
| **Cognitive EST** | | | | |
| L vmPFC | -0.15 | -0.58 | .569 | 22 |
| R vmPFC | 0.05 | 0.30 | .769 | 22 |

**Supplemental Table 5**

*Group Individual Differences*

| **Individual Difference Measure** | **vmPFC Lesion**  *M* (*SD*) | **BD Comparison**  *M* (*SD*) | **Healthy Comparison**  *M* (*SD*) | ***F*** | ***p*** | **η_p_^2^** | **Cronbach’s α** |
| --- | --- | --- | --- | --- | --- | --- | --- |
| IRI - Fantasy | 2.85 (1.05) | 3.06 (0.67) | 2.96 (0.82) | 0.19 | .829 | 0.01 | 0.78 |
| IRI – EC | 3.81 (0.65) | 3.95 (0.49) | 3.96 (0.59) | 0.28 | .759 | 0.01 | 0.69 |
| IRI – PT | 3.21 (0.91) | 3.45 (0.63) | 3.41 (0.79) | 0.32 | .726 | 0.02 | 0.82 |
| IRI – PD | 2.20 (0.61) | 2.17 (0.71) | 2.31 (0.85) | 0.15 | .863 | 0.01 | 0.82 |
| BAS – Drive | 2.50 (0.59) | 2.52 (0.54) | 2.45 (0.31) | 0.07 | .928 | 0.00 | 0.57 |
| BAS – Fun Seeking | 2.84 (0.54) | 2.65 (0.23) | 2.50 (0.53) | 1.77 | .185 | 0.09 | 0.56 |
| BAS – Reward Responsiveness | 3.25 (0.58) | 3.32 (0.28) | 3.31 (0.43) | 0.09 | .916 | 0.00 | 0.74 |
| BIS | 2.45 (0.69) | 2.88 (0.63) | 2.79 (0.47) | 1.67 | .202 | 0.09 | 0.83 |
| Self-Reported Altruism | 1.76 (0.90) | 2.14 (0.47) | 1.98 (0.46) | 1.06 | .356 | 0.06 | 0.89 |

*Note*. IRI = Interpersonal Reactivity Index; EC = Empathic Concern; PT = Perspective Taking; PD = Personal Distress. BAS = Behavioral Activation System; BIS – Behavioral Inhibition System. Due to a programming error, one item from the Self-Reported Altruism scale was not obtained from participants.

**Supplemental Table 6**

*Correlations Between Individual Difference Measures and Empathy Choice*

| **Individual Difference Measure** | **vmPFC Lesion**  *M* (*SD*) | | | | **BD Comparison**  *M* (*SD*) | | | | **Healthy Comparison**  *M* (*SD*) | | | |
| --- | --- | --- | --- | --- | --- | --- | --- | --- | --- | --- | --- | --- |
|  | *r* | *p* | 95% CI | *N* | *r* | *p* | 95% CI | *N* | *r* | *p* | 95% CI | *N* |
| **Affective EST Choice Correlations** | | | | | | | | | | | | |
| IRI - Fantasy | 0.75 | .005 | [0.28, 0.92] | 12 | 0.17 | .598 | [-0.45, 0.67] | 12 | -0.25 | .342 | [-0.66, 0.28] | 16 |
| IRI – EC | 0.79 | .002 | [0.36, 0.93] | 12 | 0.41 | .189 | [-0.23, 0.79] | 12 | 0.04 | .889 | [-0.47, 0.52] | 16 |
| IRI – PT | 0.54 | .071 | [-0.07, 0.84] | 12 | -0.73 | .007 | [-0.91, -0.23] | 12 | -0.44 | .092 | [-0.76, 0.09] | 16 |
| IRI – PD | -0.22 | .488 | [-0.70, 0.41] | 12 | 0.36 | .251 | [-0.28, 0.77] | 12 | 0.59 | .017 | [0.11, 0.83] | 16 |
| BAS – Drive | 0.43 | .163 | [-0.21, 0.80] | 12 | -0.10 | .750 | [-0.64, 0.50] | 12 | 0.38 | .145 | [-0.15, 0.73] | 16 |
| BAS – Fun Seeking | 0.20 | .549 | [-0.46, 0.71] | 11 | 0.20 | .528 | [-0.43, 0.69] | 12 | 0.35 | .180 | [-0.18, 0.72] | 16 |
| BAS – Reward Responsiveness | 0.35 | .258 | [-0.29, 0.76] | 12 | -0.09 | .784 | [-0.63, 0.51] | 12 | 0.37 | .154 | [-0.16, 0.73] | 16 |
| BIS | 0.52 | .100 | [-0.14, 0.85] | 11 | 0.62 | .031 | [0.04, 0.87] | 12 | 0.41 | .127 | [-0.14, 0.76] | 15 |
| Self-Reported Altruism | 0.61 | .045 | [-0.01, 0.88] | 11 | -0.25 | .427 | [-0.72, 0.38] | 12 | 0.32 | .224 | [-0.22, 0.70] | 16 |
| **Cognitive EST Choice Correlations** | | | | | | | | | | | | |
| IRI - Fantasy | 0.14 | .673 | [-0.48, 0.66] | 12 | 0.21 | .520 | [-0.42, 0.69] | 12 | 0.08 | .771 | [-0.44, 0.55] | 16 |
| IRI – EC | 0.01 | .965 | [-0.56, 0.58] | 12 | -0.50 | .097 | [-0.83, 0.12] | 12 | -0.08 | .766 | [-0.55, 0.43] | 16 |
| IRI – PT | 0.03 | .924 | [-0.55, 0.59] | 12 | -0.44 | .149 | [-0.80, 0.20] | 12 | -0.50 | .050 | [-0.79, 0.01] | 16 |
| IRI – PD | -0.34 | .286 | [-0.76, 0.31] | 12 | 0.09 | .780 | [-0.51, 0.63] | 12 | 0.42 | .102 | [-0.11, 0.75] | 16 |
| BAS – Drive | -0.12 | .701 | [-0.65, 0.49] | 12 | 0.41 | .185 | [-0.23, 0.79] | 12 | 0.06 | .820 | [-0.45, 0.54] | 16 |
| BAS – Fun Seeking | -0.06 | .859 | [-0.64, 0.56] | 11 | 0.17 | .600 | [-0.45, 0.67] | 12 | 0.25 | .359 | [-0.29, 0.66] | 16 |
| BAS – Reward Responsiveness | -0.39 | .213 | [-0.78, 0.26] | 12 | -0.60 | .037 | [-0.87, -0.02] | 12 | -0.05 | .841 | [-0.53, 0.45] | 16 |
| BIS | 0.07 | .837 | [-0.56, 0.64] | 11 | 0.09 | .775 | [-0.51, 0.63] | 12 | 0.55 | .035 | [0.03, 0.82] | 15 |
| Self-Reported Altruism | -0.03 | .935 | [-0.62, 0.58] | 11 | -0.47 | .121 | [-0.82, 0.16] | 12 | -0.18 | .511 | [-0.61, 0.35] | 16 |

**Supplemental Table 7**

*Comparisons of Correlation Magnitudes Between Individual Difference Measures and Empathy Choice Proportion*

|  | **vmPFC Lesion Group** | | | |
| --- | --- | --- | --- | --- |
|  | versus Brain-Damaged Comparison | | versus Healthy Comparison | |
| **Affective EST** | **Z** | ***p*** | **Z** | ***p*** |
| IRI - Fantasy | 1.70 | .089 | 2.83 | .005 |
| IRI – EC | 1.35 | .177 | 2.38 | .017 |
| IRI – PT | 3.25 | .001 | 2.48 | .013 |
| IRI – PD | -1.27 | .203 | -2.09 | .038 |
| BAS – Drive | 1.19 | .235 | 0.14 | .890 |
| BAS – Fun Seeking | 0.00 | 1.000 | -0.36 | .717 |
| BAS – Reward Responsiveness | 0.97 | .334 | -0.05 | .958 |
| BIS | -0.31 | .760 | 0.31 | .758 |
| Self-Reported Altruism | 1.98 | .047 | 0.84 | .401 |
| **Cognitive EST** | **Z** | ***p*** | **Z** | ***p*** |
| IRI - Fantasy | -0.15 | .878 | 0.14 | .889 |
| IRI – EC | 1.87 | .235 | 0.21 | .835 |
| IRI – PT | 1.07 | .287 | 1.34 | .182 |
| IRI – PD | -0.94 | .346 | -1.85 | .065 |
| BAS – Drive | -1.18 | .238 | -0.42 | .677 |
| BAS – Fun Seeking | -0.48 | .633 | -0.70 | .483 |
| BAS – Reward Responsiveness | 0.60 | .551 | -0.83 | .404 |
| BIS | -0.04 | .967 | -1.20 | .230 |
| Self-Reported Altruism | 0.99 | .323 | 0.34 | .735 |

1. The number of emotion words used was associated with choosing empathy in the affective EST across the sample: *r*_PositiveEmotions_ = 0.41, *p* = .007; *r*_NegativeEmotions_ = 0.31, *p* = .045.
